# Sustainable and Surfactant-Free Synthesis of Negatively Charged Acrylamide Nanogels for Biomedical Applications

**DOI:** 10.1021/acs.macromol.4c02128

**Published:** 2025-01-23

**Authors:** Davide Mazzali, Gabriela Rath, Alexander Röntgen, Vaidehi Roy Chowdhury, Michele Vendruscolo, Marina Resmini

**Affiliations:** † Department of Chemistry, SPCS, Queen Mary University of London, London E1 4NS, U.K.; ‡ Centre for Misfolding Diseases, Yusuf Hamied Department of Chemistry, University of Cambridge, Cambridge CB2 1EW, U.K.

## Abstract

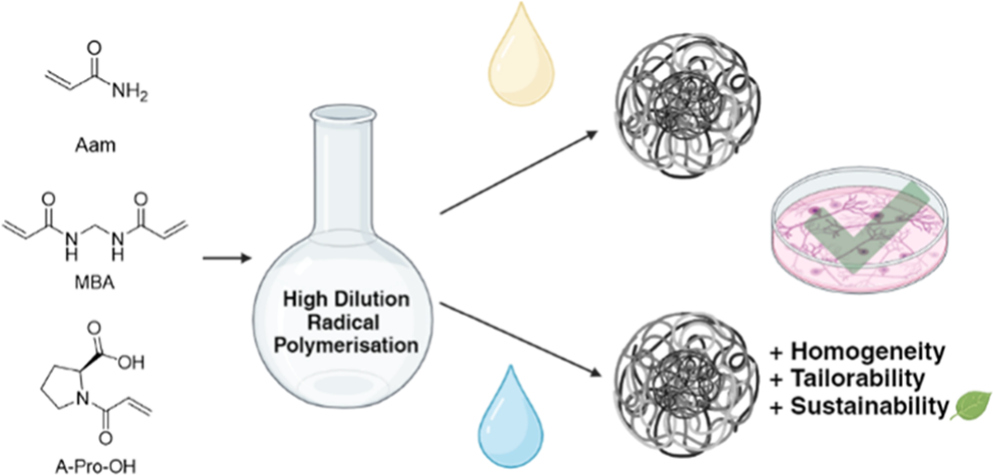

Nanogels offer unique advantages, like high surface-to-volume ratio, scalable synthetic methods, and easily tailored formulations, that allow us to control size and introduce stimuli-responsive properties. Their potential for drug delivery is significant due to their biocompatibility, high drug loading capacity, and controlled and sustained drug release. The development of greener and sustainable processes is essential for large-scale applications. We report the synthesis in water of covalently cross-linked acrylamide-based nanogels, both neutral and negatively charged, with varying amounts of acryloyl-l-proline, using high-dilution radical polymerization, without the need for surfactants. The use of a water-based synthesis resulted in nanogels with high monomer conversions and chemical yields, as well as lower polydispersity and smaller particle sizes for the negatively charged nanogels, leading to a more efficient synthetic methodology, with reduced loss of starting materials, higher potential for scalability, and reduction in costs. The suitability of these nanogels for biomedical applications was supported by cytotoxicity studies showing no significant reduction in viability on a human neuroblastoma cell line.

## 1 Introduction

Innovative drug delivery systems are key in drug discovery and development, as they enable the effective administration of therapeutic agents. Advances in the field of nanomaterials contribute to addressing some of the challenges in drug delivery, such as the off-target toxicity, low loading capacity, and poor solubility of active molecules,^1−3^ with controlled release^4,5^ and permeation through biological barriers^6^ playing important roles. Among the systems being investigated are liposomes,^7^ polymeric nanoparticles (NPs),^8^ micelles,^9^ and covalently cross-linked (XL) nanogels (NGs), with the latter offering great potential.^10^ NGs are characterized by small size (10–100 nm) and high drug loading capacity, while their formulation can be easily tailored to introduce stimuli-responsiveness to triggers such as infrared radiation,^11^ oxidation,^12^ temperature,^13^ and pH,^14,15^ an attractive feature for drug delivery applications.^16−19^

The most common synthetic strategy for the preparation of NGs is radical polymerization (RP),^20^ either controlled^21−40^ or free, either in heterogeneous or homogeneous phase. Control of the molecular weight during the synthesis is a priority for all NG applications, given the established correlation between the size and properties of the isolated polymers.^24^ Heterogeneous polymerization techniques such as (inverse) emulsion/microemulsion/miniemulsion^25−27^ or precipitation/dispersion^28^ require surfactants to stabilize the growing polymeric chains and control the final particle size. The use of surfactants and emulsifiers, although potentially advantageous, requires additional purification steps that can affect the drug loading as well as the nanoparticles’ stability, increase potential issues of toxicity in vivo,^29^ and make the scalability of the process more laborious. High-dilution radical polymerization (HDRP) is a homogeneous polymerization method that does not require surfactants to obtain colloidally stable nanogels and uses monomer concentrations to avoid macrogelation while controlling polymer weight.^30^ HDRP can be optimized to lead to high monomer conversions and yields, good control of particle size, and easy tailoring of the formulation with the addition of different functional monomers and cross-linkers.

We used HDRP in organic solvents for the synthesis of NGs for applications in catalysis,^31^ sensing,^32^ and drug delivery,^33^ focusing on the relationship between formulation and properties, their interaction with biomolecules in vitro,^34^ and the effect of different synthetic procedures.^35^ As interest toward NGs continues to grow, especially in the biomedical field, issues around the use of organic solvents and sustainability of the process are becoming prominent. Studies of the effects of changing the solvent system include the synthesis of core–shell nano/microgels with poly(ethylene glycol) chains using surfactant-free emulsion polymerization in water,^36^ the impact of changing water-to-ethanol ratio in a cosolvent system,^37^ using biobased synthesis-solvents,^38^ and the effect of solvents on the swelling ability of bulk hydrogels.^39^

HDRP has been extensively employed for the synthesis of NGs using organic solvents as the polymerization medium, thanks to the versatility they offer in terms of starting monomers that they can solvate.^41,42^ However, this has limited any upscaling, given the large volumes of organic solvents required. The use of water could improve the sustainability of the overall method. Albeit advantageous, this approach has not been explored extensively, and in particular, the impact that this change would have on the morphology of the particles. In this work, we report the green and surfactant-free synthesis in water and characterization of acrylamide-based NGs, covalently cross-linked (XL) with 20 mol % of *N*,*N*′-methylenebis(acrylamide) (MBA), neutral or negatively charged by the addition of varying amounts of the negatively charged acryloyl-l-proline (A-Pro-OH). Monomer conversions were determined by proton nuclear magnetic resonance (^1^H NMR) spectroscopy, while size, polydispersity, and surface charge were quantified by using dynamic light scattering (DLS). All data were compared to the same formulations synthesized in dimethyl sulfoxide (DMSO) to evaluate the effects of changing solvent on properties and morphology. The cytotoxicity of NGs was also evaluated in SH-SY5Y (human neuroblastoma) cells, given the potential applications of NGs for drug delivery.

## 2 Materials and Methods

### 2.1 Materials

*N*,*N*′-Methylenebis(acrylamide) (MBA, 99%), acrylamide (Aam, 98%), 1,2,4,5-tetramethylbenzene (TMB, 98%), *N*,*N*,*N*′,*N*′-tetramethylethylenediamine (TEMED, 99%), potassium persulfate (KPS, ≥99.0%), sodium phosphate dibasic heptahydrate, and sodium phosphate monobasic heptahydrate were purchased from Sigma-Aldrich (Gillingham, U.K.) and employed as received. 2,2′-Azobis(2-methylpropionitrile) (AIBN, 98%) was purchased from Sigma-Aldrich (Gillingham, U.K.) and used after recrystallization in methanol. Dry dimethyl sulfoxide (DMSO, 99%) was purchased from Goss Scientific (Crewe, U.K.), and deuterated dimethyl sulfoxide (DMSO-*d*_6_, 99%) was purchased from Cambridge Isotope Laboratories (Cambridge, U.K.). Acryloyl-l-proline (A-Pro-OH) was synthesized using a previously reported procedure.^43^ Dialysis membranes (molecular weight cutoff 3.5 kDa) were purchased from Medicell International Ltd (London, U.K.). Poly(ether sulfone) (PES) syringe filters with pore sizes of 0.2 μm were obtained from Fisher Scientific (Loughborough, U.K.). The 3-(4,5-dimethylthiazol-2-yl)-2,5-diphenyltetrazolium bromide (MTT) assay kit was purchased from Abcam (Cambridge, U.K.), and Dulbecco’s modified Eagle’s medium (DMEM/F-12), GlutaMAX supplement, Roswell Park Memorial Institute (RPMI) cell medium 1640, fetal bovine serum (FBS), trypsin–ethylenediaminetetraacetic acid (EDTA) (0.25%), and Dulbecco’s phosphate-buffered saline (DPBS–MgCl_2_–CaCl_2_) were purchased from Fisher Scientific (Loughborough, U.K.).

### 2.2 Synthesis of NGs in DMSO

For the standard preparation containing Aam and MBA in the ratio 80:20 (mol %), the calculated quantities of Aam (backbone monomer), MBA (cross-linker), and initiator (AIBN) were weighed according to each formulation and dissolved in dry DMSO in a round-bottom flask (RBF). The volume of DMSO was calculated to obtain a 1% (w/w) final monomer concentration. AIBN (initiator) was added to the RBF at a concentration of 1% of the total moles of double bonds in the reaction mixture, after which the RBF was sealed and purged with nitrogen. The vessel was then placed in an oil bath at 70 °C for 24 h. 100 μL of samples to quantify monomer conversions were taken at the start (*t* = 0 h) and at the end (*t* = 24 h) of the reaction.

### 2.3 Synthesis of NGs in Water

The synthesis of NGs in water followed the same procedure described in Section 2.2, with the moles of KPS and TEMED calculated to be 1 and 5% of the total moles of double bonds, respectively. To prevent the reaction from starting prematurely, these were weighed and solubilized in a separate RBF, which was also purged with nitrogen. The concentration of the initiator stock solution was determined so that 1 mL of solution would be added to the reaction mixture to bring its final concentration to 1% (w/w) (comparable to that of the DMSO synthesis). After adding the initiator solution, the reaction mixture was placed in a preheated oil bath at 30 °C. 100 μL of samples to quantify monomer conversions were taken at the start (*t* = 0 h) and at the end (*t* = 24 h) of the reaction.

### 2.4 Purification and Isolation of NGs

After polymerization, NGs were dialyzed against deionized water for at least 48 h, changing water every 8 h, using membranes with a 3.5 kDa molecular cutoff. Purified NGs were freeze-dried for at least 2 days and stored at room temperature (RT) until use.

### 2.5 Monomer Conversion via ^1^H NMR Spectroscopy

Monomer conversion was evaluated by ^1^H NMR spectroscopy for each formulation at *t* = 0 and 24 h. TMB was used as the internal standard (IS) at a final concentration of 20 mg/mL in DMSO-*d*_6_, with total volumes of 500 μL. ^1^H NMR spectra were acquired in a solvent suppression mode at 298 K using a Bruker AV III 400 spectrometer (400 MHz). Spectra were processed with TopSpin software (version 4.2.0, Bruker) to quantify the signal of each monomer at *t* = 0 and 24 h (Aam at δ = 6.04 ppm, A-Pro-OH at δ = 6.61 ppm, and MBA at δ = 5.63 ppm) by integrating it against the peak of the acrylic protons of the IS (δ = 6.87 ppm).

### 2.6 Particle Size Measurement by Dynamic Light Scattering

NG samples for DLS were prepared by dispersing about 3 mg of the isolated, freeze-dried polymer in phosphate buffer (PB) 10 mM pH 7.4 at a final concentration of 1 mg/mL. The solution was sonicated for 10 min and filtered using a 0.2 μm PES filter directly to a disposable plastic precleaned cuvette (Fisher Scientific, Leicestershire, U.K., catalogue no. 15520814) to minimize dust contamination. Samples were analyzed in triplicate at 25 °C using a Malvern Zetasizer Ultra. Backscatter (173°) angle mode was used to determine the size distribution and polydispersity index (PDI) for each sample. Data were processed using software ZX Xplorer (version 2.2.0.147, Malvern Panalytical Ltd., Malvern, U.K.) and plotted as mean values ± standard deviation (SD), using GraphPad Prism 10.0.0.

### 2.7 ζ-Potential Analysis

NG samples for DLS were prepared by dispersing about 3 mg of dry polymer in phosphate buffer (PB) 10 mM pH 7.4 at a final concentration of 1 mg/mL. The solution was sonicated for 10 min and filtered using a PES filter 0.2 μm directly to a disposable folded capillary cell (1080, Malvern Panalytical Ltd., Malvern, U.K.). To minimize dust contamination, the capillary cell was flushed with compressed air and washed with PB immediately before samples were transferred. Samples were analyzed in triplicate at 25 °C using a Malvern Zetasizer Ultra.

### 2.8 Acute Cytotoxicity Studies: SH-SY5Y Cells

Human SH-SY5Y neuroblastoma cells were cultured in DMEM/F-12, GlutaMAX supplemented with 10% (v/v) FBS on 75 cm^2^-treated polystyrene flasks (Greiner Bio-One, Stonehouse, U.K). Cells were grown at 37 °C in a 5% CO_2_-humified atmosphere and split at an 80% confluency. Cell viability was measured using an MTT assay. Cells were seeded on 96-well plates (Greiner Bio-One, Stonehouse, U.K) at a density of 10,000 cells/well in 100 μL of cell medium. After incubation for 24 h at 37 °C, the medium was replaced with either fresh medium (medium control), 10% (v/v) ultrapure water in fresh medium (vehicle control), or nanoparticles in 10% (v/v) ultrapure water in fresh medium. Cells were treated in quintuplicate and incubated for another 24 h at 37 °C. After that, the medium was discarded, and cells were incubated with 0.5 mg/mL of MTT in RPMI medium for 4 h at 37 °C. The solution was discarded, and the formazan product was solubilized by incubation in cell lysis buffer (100 μL per well) at 500 rpm and 37 °C for 15 min on a PHMP Grant-Bio Thermoshaker. Absorbance at λ = 570 nm was measured on a CLARIOStar plate reader (BMG Labtech, Aylesbury, U.K.), and the cell viability was normalized to the medium control.

### 2.9 Transmission Electron Microscopy (TEM)

Nanogels were imaged using transmission electron microscopy (TEM), both in dry state and in cryogenic mode. Briefly, 8 μL of each reconstituted solution was deposited on a copper grid (Agar Scientific, Rotherham, U.K.), blotted, and stained with a 1% solution of uranyl acetate in water. For cryogenic-TEM (cryo-TEM), 4 μL of each solution was deposited on a copper grid (Agar Scientific, Rotherham, U.K.), blotted, and immediately frozen by plunging in a liquid ethane bath. The samples were imaged using a JEM-F200 microscope (200 kV, JEOL Ltd., Tokyo, Japan). ImageJ (v.1.54) was used to analyze and measure the size of the particles.

### 2.10 Data Analysis

Data visualization and analysis (monomer conversion, chemical yield (CY), DLS measurements, and cell viability) were performed using GraphPad Prism 10.0.0, plotting mean values ± SD, unless otherwise specified. Data were analyzed for normal distribution using the Shapiro–Wilk test. Two-way analysis of variance (ANOVA) with Šídák’s multiple comparison test was used to analyze the monomer conversion and chemical yield, while one-way ANOVA was used for DLS data. Multiple comparison Student’s *t*-test was used to analyze polydispersity values. The Mann–Whitney test was used to analyze size values obtained from TEM imaging.

## 3 Results and Discussion

### 3.1 Synthesis and Characterization of NGs: Monomer Conversion and Chemical Yield

**Figure 1:**
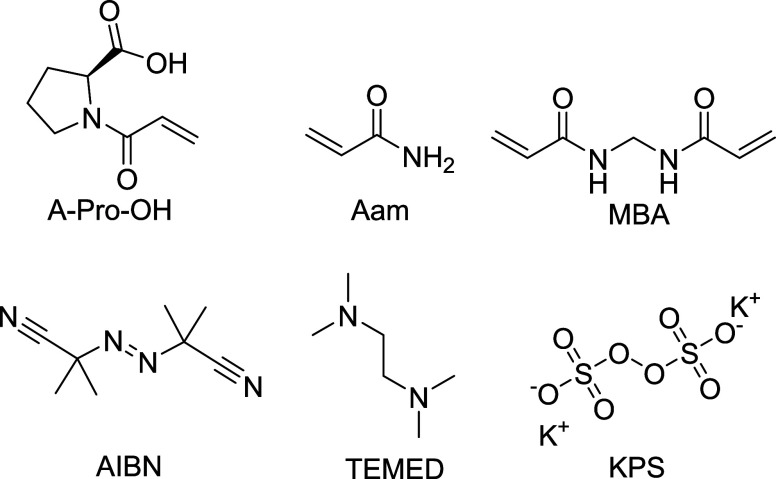
Chemical structures of monomers, cross-linker and initiators used to synthesize the NGs.

A small library of 10 NGs was synthesized via HDRP following our previously reported protocol^35^ using DMSO or water as the solvent (Table 1) and without the addition of any surfactants. DMSO was chosen as the medium due to its excellent solvating power for hydrophilic substrates and its moderate toxicity. Aam (Figure 1) was chosen as the backbone monomer given its solubility in both solvent systems (DMSO and water). The cross-linker (MBA) content was fixed at 20 mol %, as this amount was found to be optimal for drug loading^33^ and could provide a good balance between the flexibility of the matrix and the three-dimensional structure of the NG. A-Pro-OH was added as a functional monomer designed to impart a negative charge to the NGs, introducing a potentially pH-responsive trigger.

**Table 1:** Chemical Composition of the Nanogels Synthesized for This Study^a^

formulation	synthetic solvent	Aam (% mol/mol)	MBA (% mol/mol)	A-Pro-OH (% mol/mol)
NG1	DMSO	80	20	0
NG2		77.5		2.5
NG3		75		5
NG4		70		10
NG5		65		15
NG6	water	80	20	0
NG7		77.5		2.5
NG8		75		5
NG9		70		10
NG10		65		15

a All the formulations were synthesized in triplicates.

The A-Pro-OH monomer content in the formulations was varied between 2.5 and 15 mol % to evaluate the impact of the negative charge on the morphology and the cytotoxicity of the NGs. For all preparations, the total monomer concentration (*C*_m_) was kept to 1% (w/w), using AIBN as the initiator for the NGs prepared in DMSO, while KPS/TEMED was used for the formulations synthesized in water, given AIBN’s limited solubility in this solvent.^44^ The preparation of NGs in both media was justified by the objective of evaluating how the change to a more sustainable and greener solvent could affect the NG morphology and properties. All of the formulations were prepared in triplicate to ensure the reproducibility of the synthetic procedure.

**Figure 2:**
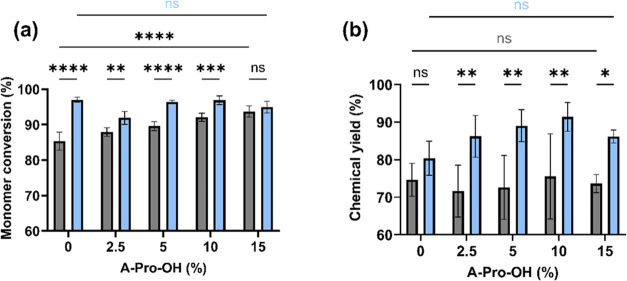
Total monomer conversion (a) and chemical yields (CY) (b) for nanogels synthesized in DMSO (gray) and water (light blue). Data are presented as mean values ± SD (*n* = 3), *p < 0.01, **p < 0.001, *** 0.001 < *p* < 0.0001, and ****p < 0.0001, ns = non significant.

Given the random nature of radical polymerization, any evaluation of the solvent effect on the NGs required evidence of consistency between formulation and final chemical composition of the isolated polymers. For this reason, monomer conversions (determined by ^1^H NMR; Figure S1) and chemical yields were quantified (Figure 2).

The synthetic procedure yielded high total monomer conversions for all of the formulations tested (Figure 2a; MC_t_ > 80%). However, the neutral NG synthesized in water had a significantly (*p* < 0.0001) higher MC_t_ than the one obtained in DMSO, as a result of the different reactivities of Aam in the alternative solvent systems.^45,46^ Aam is fully solvated and protonated in water, which reduces the rate of bimolecular chain termination (via electrostatic interactions) and destabilizes the unpaired electron, leading to a higher reactivity. In contrast, Aam exists mainly as a dimer/trimer in DMSO due to its reduced ability to form H-bonds with the solvent, which leads to a slower propagation step of the polymerization.^47^ This difference was confirmed by the evaluation of the monomer conversions for the individual components (MC_s_), showing that the reaction for Aam never exceeded 90% (Table S1). When the negatively charged functional monomer A-Pro-OH was introduced in the formulation, the MC_t_ for all DMSO NGs increased (Figure 2a), even at the lowest concentration of 2.5 mol %. This result is not surprising, given that the A-Pro-OH monomer was previously shown to have a higher reactivity than Aam in DMSO.^48^ This effect increased steadily with increasing A-Pro-OH molar content, up to 15 mol %, providing evidence of the positive impact on MC_t_ of the functional monomer in the formulation. At the highest A-Pro-OH concentration, no significant difference (ns, *p* = 0.30) was observed in MC_t_ between formulations prepared in DMSO and water. In the case of NGs synthesized in water, the addition of the negatively charged functional monomer did not impact the formulations, with no significant difference (ns, *p* = 0.99) in MC_t_ between neutral and negatively charged NGs.

The change in the polymerization solvent from DMSO to water had a remarkable effect on the chemical yields (Figure 2b). As opposed to the MC_t_ data, the choice of solvent did not significantly (ns, *p* = 0.26) impact the chemical yield of neutral NGs; however, when the functional monomer A-Pro-OH was added, the chemical yields for the water NGs were consistently much higher than for DMSO NGs. This is particularly interesting given that all of the polymerizations were carried out at the same total monomer concentration (1%). At the lowest concentration of A-Pro-OH (2.5 mol %), there was already a significant difference (*p* < 0.001) in chemical yields obtained between the same formulation in water vs DMSO due to the lower reactivity of Aam in the latter.^47^ This effect became more significant (*p* < 0.001) when higher amounts of functional monomer were added to the formulation (up to 15 mol %). However, the chemical yields of the negatively charged NGs prepared in water were not impacted by the increased amount of functional monomer. The differences observed between water and DMSO could be the result of not only variations in particle size but also polydispersity of the preparations, hence requiring further characterization.

### 3.2 *D*_h_ and ζ-Potential Analysis via DLS

**Figure 3:**
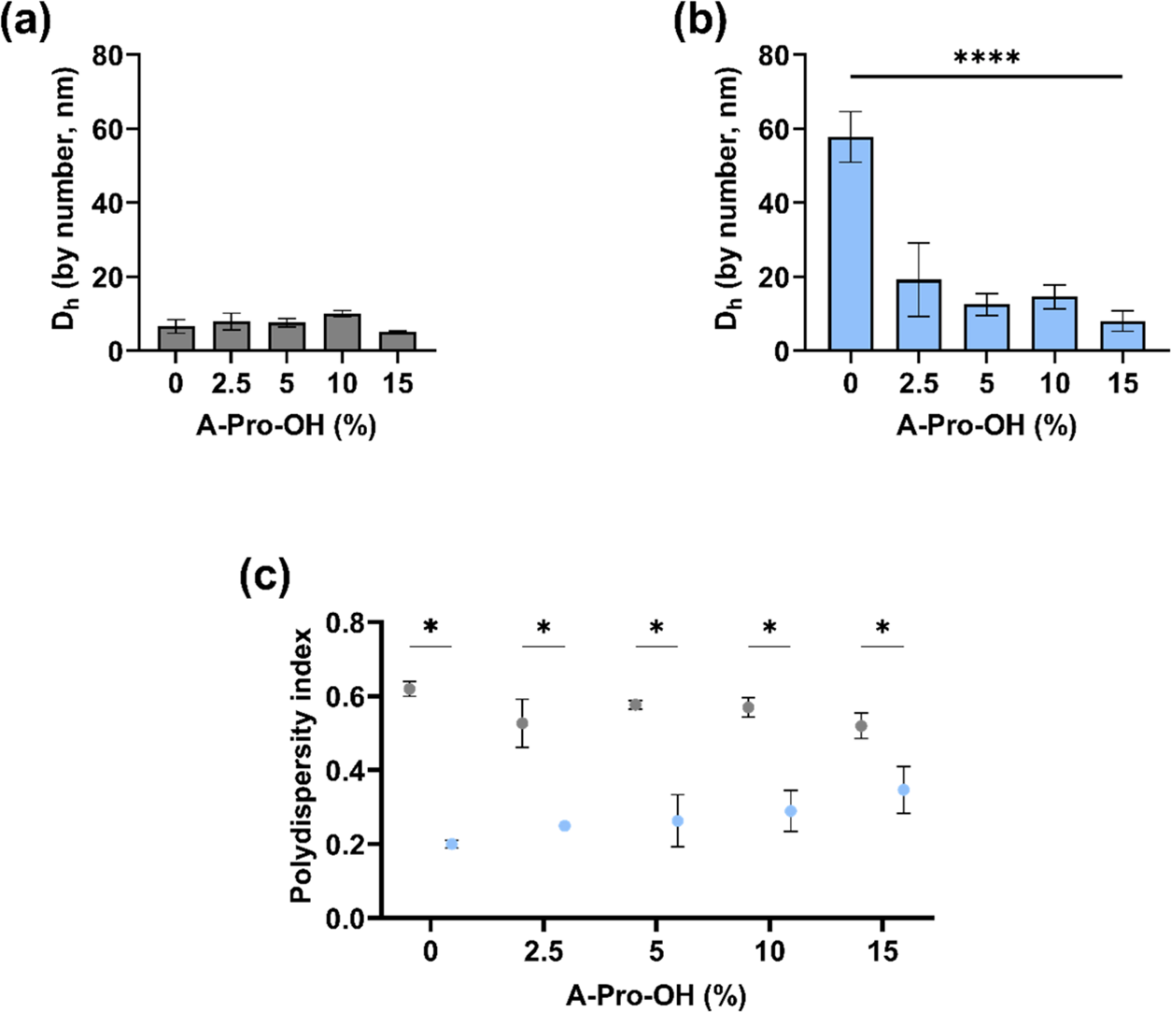
Hydrodynamic diameter measured by DLS for nanogels synthesized in DMSO (a) and water (b). (c) Polydispersity index calculated by DLS for the same formulations (water—light blue, DMSO—gray) in phosphate buffer (PB) 10 mM pH 7.4. Data are presented as mean values ± SD (*n* = 3), *p < 0.01, ****p < 0.0001.

DLS measurements were carried out to analyze the particle sizes with NG solutions in phosphate buffer (10 mM, pH 7.4), forming stable colloidal solutions. The measurements (Figure 3a) showed that NGs synthesized in DMSO resulted in particles with a hydrodynamic diameter (*D*_h_) less than 10 nm (Figure S2) for both neutral and negatively charged particles, which is consistent with previously reported data.^49^ Polymerization in DMSO with CM_t_ = 1% (w/w) is affected by the solvent limiting the reactivity of Aam,^47^ stabilizing the growing polymeric chains and leading to smaller particles. In addition, when the functional monomer A-Pro-OH was introduced in the formulation, the size was not impacted, regardless of its concentration. All of the nanogels showed an average *D*_h_ of around 10 nm.

For the NGs synthesized in water (Figure 3b), the data show that the neutral NG has a much larger particle size (around 58 nm; Figure S3) compared to the same formulation prepared in DMSO; however, when the charged monomer was introduced, the resulting polymer was considerably smaller, around 20 nm. The presence of the ionized carboxylic moiety in the A-Pro-OH monomer is likely to result in electrostatic repulsion leading to the formation of smaller particles, even when present in concentrations as low as 2.5 mol %. Interestingly, no significant differences were observed for formulations with different A-Pro-OH contents, suggesting that this effect is not concentration-dependent.

The polydispersity index (PDI; Figure 3c) for all of the preparations was determined from the DLS data, and the values displayed an interesting trend. The NGs synthesized in water had significantly lower (*p* < 0.01) PDI values than NGs synthesized in DMSO. The introduction of A-Pro-OH did not significantly impact the PDI values of both DMSO and water NGs for all concentrations tested. The data provide evidence that helps to interpret the solvent effect on the chemical yields. The higher PDI values in DMSO suggest that the polymerization results in a much higher proportion of smaller particles that are then lost during the purification step by dialysis. High polydispersity has been shown to lead to a loss of colloidal stability when the NG synthesis is scaled up,^50^ which provides an additional advantage, to the choice of using water as a better solvent system.

**Figure 4:**
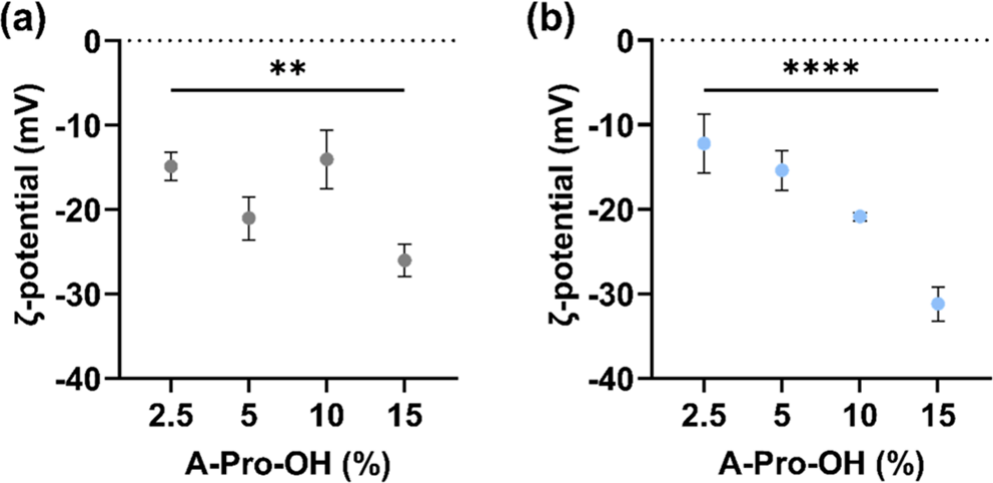
ζ-Potential of different formulations in water (a) and in DMSO (b) measured in PB 10 mM pH 7.4. Data are presented as mean values ± SD (*n* = 3), **p < 0.01, and ****p < 0.0001.

The ζ-potential values of all formulations were measured by DLS (Figure 4) to provide further data on the physicochemical properties of the polymers. NGs with 2.5 mol % of A-Pro-OH showed similar ζ-potential values (around −15 mV), regardless of the solvent used during synthesis. On the other hand, water NGs with 15 mol % of A-Pro-OH showed significantly (*p* < 0.0001) different surface charges (∼−30 mV) compared to DMSO (−25 mV). Interestingly, the NGs produced in water showed a negative correlation between the A-Pro-OH content and total surface charge, which cannot be observed for the NGs prepared in DMSO.

The data obtained with DLS and ^1^H NMR together with the ζ-potential values provide evidence of the effect that the solvent used during the polymerization has on the morphology and properties of the NGs. When prepared in water, the ionized A-Pro-OH monomer is more likely to be localized near the surface of the NG, where it can minimize the electrostatic repulsion. The use of the KPS/TEMED pair for the initiation leads to the presence of tertiary amine end-groups in the NG network,^51^ potentially impacting the surface charge quantification; however, no significant evidence of this was found in this case, even at low concentrations of A-Pro-OH. When prepared in DMSO, the localization of the monomers in the NG structure is influenced by their reactivity, with both A-Pro-OH and MBA having a similar and faster reactivity compared to AAm,^48,52,53^ resulting in a more peripheric incorporation of Aam in the particle structure. This is further confirmed by the lack of a trend in ζ-potential values with increasing A-Pro-OH content for the NGs prepared in DMSO.

### 3.3 Transmission Electron Microscopy

**Figure 5:**
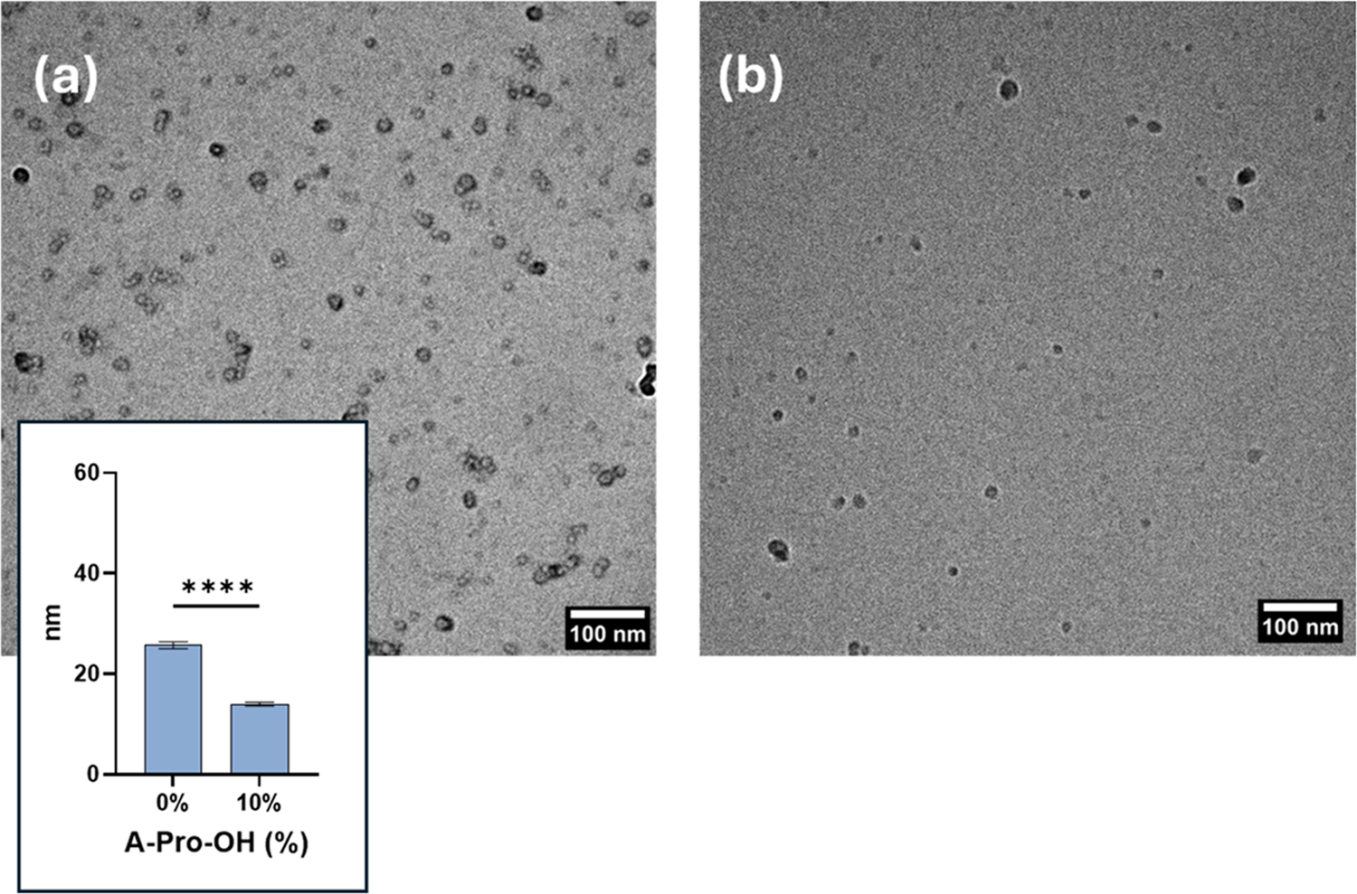
Cryo-EM images of nanogels synthesized in water with no functional monomer (a) and 10% A-Pro-OH (b). Data are presented as mean values ± standard error of the mean (SEM) (*n* = 105, ****p < 0.0001).

The images obtained via transmission electron microscopy (TEM) reinforced the results obtained by DLS on the size of the particles and the effect of the introduction of the A-Pro-OH monomer in water. Overall, the introduction of the negatively charged monomer reduced the average diameter of the NGs (Figure 5).

The size measured by cryo-TEM is consistent with that observed by DLS, although the diameters observed are smaller. This is explained by the intrinsic DLS being a technique based on scattering, which provides the hydrodynamic diameter of the particles measured, as opposed to electron microscopy, which provides a direct result on the particle diameter. A similar reduction in size is shown by negative staining TEM (Figure S5) for nanogels produced in DMSO, which contrasts with DLS results. In this case, one of the possible explanations is that the introduction of A-Pro-OH counteracts the loss of a tridimensional structure due to the drying effect.

Overall, the TEM data indicate that the negatively charged NGs containing A-Pro-OH show different properties when synthesized in water rather than in DMSO. HDRP in water leads to better control over particle size and surface charge, as well as lower polydispersity, regardless of the amount of functional monomer introduced. These results highlight how the HDRP process can be tailored to a more sustainable, scalable, and controllable approach. Further, it can support the development of negatively charged nanogels for a variety of applications, including drug delivery.

### 3.4 Acute Cytotoxicity Assay

**Figure 6:**
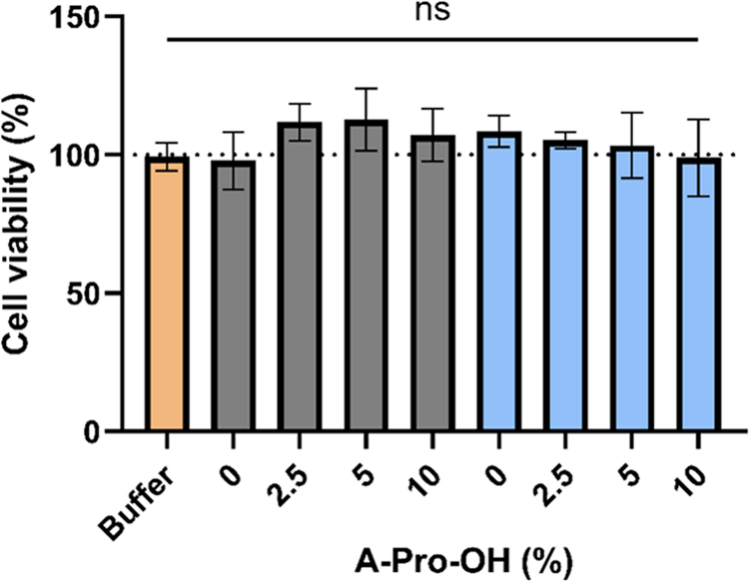
Cell viability expressed as the percentage of medium control for different nanogels containing 0–10 mol % A-Pro-OH (vehicle control in orange, DMSO in gray, water in light blue). Concentration 1 mg/mL. Data are presented as mean ± SD (*n* = 3). The changes in cell viability are not statistically significant under any of the conditions tested.

Biocompatibility is paramount when developing new materials for drug delivery applications. Therefore, it is important to test the cytotoxicity of the NGs and evaluate if the choice of the synthetic solvent and changes in morphology and properties have any impact on how these nanoparticles interact with bioentities. For this study, we evaluated the cytotoxicity of the NGs containing up to 10 mol % of A-Pro-OH on the SH-SY5Y human neuroblastoma cell line via an MTT assay, as it is a standard and reproducible cell line,^54^ to evaluate a potential application of the NGs to drug delivery in cellular systems. Cells were incubated with 0.1 and 0.5 mg/mL (Figure S4) and 1 mg/mL (Figure 6) of NGs synthesized in DMSO or in water. Overall, the data show that all of the formulations proved to be nontoxic even at high concentrations, regardless of the A-Pro-OH content or the synthetic solvent used. The data suggest that purification steps were effective in both synthetic methods and that the morphological differences introduced by the different solvents do not play a significant role in the biocompatibility in vitro. These data are coherent with reports on other cell lines regarding negatively charged NGs^27,55^ and pave the way for further studies in vivo.

## 4 Conclusions

This study evaluated a greener and sustainable synthetic approach for the synthesis in water of neutral and negatively charged Aam-based NGs via HDRP, without the use of surfactants, and how the change in solvent impacted the morphology and properties of the nanoparticles. The data that we reported provide evidence that water-based synthesis leads to NGs with higher monomer conversions and chemical yields, resulting in very good consistency between the formulation and final composition after isolation of the NGs. The polymerization in water led to a larger particle size for the neutral NG. The introduction of the A-Pro-OH monomer resulted in a smaller diameter due to the electrostatic repulsion with the growing polymer chains, confirmed by both scattering and electronic microscopy techniques; it also significantly reduced the polydispersity of the colloidal solutions. This translates into a more efficient synthetic methodology with reduced loss of starting materials, higher potential for scalability, and reduction in costs. In addition, cytotoxicity studies were carried out on the NGs, which demonstrated no significant reduction in viability on a human neuroblastoma cell line, demonstrating the safety of the nanoparticles and providing a solid base upon which to build for potential applications in biomedicine.

Future applications of nanomaterials as drug delivery systems will be significantly impacted by their ability to develop scalable synthetic approaches. In this context, the removal of organic solvents will be key to obtaining green and sustainable methodologies. The results that we reported demonstrate that water can be utilized successfully as a solvent for the synthesis of negatively charged NGs without the use of surfactants, consistently leading to particles with low polydispersity, tailored surface charge, and small particle size.

## Supporting Information Available

Nanogel characterization data (equations, isolated chemical yield values, and ^1^H NMR monomer conversion of the replicates), DLS data (size distribution plots and correlograms), TEM micrographs (negative staining and cryogenic), and MTT assay results (lower concentrations) (PDF)
