# Supplementary material for: Sustainable and Surfactant-Free Synthesis of Negatively Charged Acrylamide Nanogels for Biomedical Applications
Source: Macromolecules. 2025 Jan 23;58(3):1206–13. doi: 10.1021/acs.macromol.4c02128 (PMC11823596; doi:10.1021/acs.macromol.4c02128)

Supporting Information: Sustainable and Surfactant-Free Synthesis of Negatively Charged Acrylamide Nanogels for Biomedical Applications

Davide Mazzali[a], Gabriela Rath[a], Alexander Röntgen[b], Vaidehi Roy Chowdhury[b], Michele Vendruscolo[b], Marina Resmini[a*]

[a] Department of Chemistry, SPCS, Queen Mary University of London, London E1 4NS, UK;

[b] Centre for Misfolding Diseases, Yusuf Hamied Department of Chemistry, University of Cambridge, Cambridge CB2 1EW, UK.

Supplementary Tables

Feed compositions for all nanogels: moles (n) of comonomer/crosslinker, initiators (AIBN and KPS/TEMED depending on the formulation), and volume (V) of solvent, were calculated using the following equations:

$$n_{\text{comonomer/crosslinker}} = n_{\text{Aam}} \times \frac{comonomer\%}{Aam\%} \qquad \textit{(Eq. S1)}$$

$$n_{\text{initiator}} = (n_{\text{Aam}} + n_{\text{A-Pro-OH}} + 2n_{\text{MBA}}) \times \%initiator \qquad \textit{(Eq. S2)}$$

$$V_{\text{solvent}} = \left(\frac{mass_{\text{TOTALmomomers}} \times (1 - C_{\text{M}})}{C_{\text{M}}}\right) \times \frac{1}{\rho_{\text{solvent}}} \qquad \textit{(Eq. S3)}$$

where C_{M} = total monomer concentration (%*w/w*). The recalculated final composition for each monomer was obtained with the following equation:

$$recalculated\ composition = MCr \cdot \frac{n_{\text{w}}}{n_{\text{Aam}} + n_{\text{MBA}} + n_{\text{A-Pro-OH}}} \qquad \textit{(Eq. S4)}$$

Where MCr is the relative monomer conversion, n_{W} is the number of moles weighed for the selected monomer in the composition, and the denominator is the total sum of moles converted by the reaction.

Table S1. Relative monomer conversion (MC_r) and recalculated composition for each of the triplicates of the nanogel formulations produced in DMSO (NG 1-5).

Preparation	Relative monomer conversion (MC_r)			Recalculated compositions		
	Aam (%)	MBA (%)	A-Pro-OH (%)	Aam (%)	MBA (%)	A-Pro-OH (%)
NG1	85.7	96.4	-	78.1	21.9	-
	79.4	96.7	-	76.7	23.3	-
	82.6	97.0	-	77.3	22.7	-
NG2	84.6	>99.9	>99.9	74.5	22.7	2.8
	85.9	>99.9	>99.9	74.7	22.5	2.8
	86.6	>99.9	>99.9	75.0	22.2	2.8
NG3	85.9	>99.9	>99.9	72.1	22.3	5.6
	86.0	94.6	>99.9	73	21.4	5.6
	87.9	>99.9	>99.9	71.1	23.1	5.8
NG4	90.5	>99.9	>99.9	67.8	21.5	10.7
	88.6	>99.9	>99.9	67.4	21.7	10.9
	87.1	>99.9	>99.9	67.0	22.1	10.9
NG5	92.3	>99.9	>99.9	63.4	`20.9	15.7
	88.2	>99.9	>99.9	62.1	21.7	16.2
	89.9	>99.9	>99.9	62.6	21.4	16.0

Table S2. Relative monomer conversion (MC_r) and recalculated composition for each of the triplicates of the nanogel formulations produced in water (NGs 6-10).

Preparation	Relative monomer conversion (MC_r)			Recalculated compositions		
	Aam (%)	MBA (%)	A-Pro-OH (%)	Aam (%)	MBA (%)	A-Pro-OH (%)
NG6	96.5	>99.9	-	79.4	20.6	-
	97.0	>99.9	-	79.5	20.5	-
	95.2	>99.9	-	79.2	20.8	-
NG7	89.4	>99.9	>99.9	75.5	21.8	2.7
	91.9	>99.9	>99.9	76.0	21.3	2.7
	84.6	>99.9	>99.9	74.5	22.7	2.8
NG8	95.2	>99.9	>99.9	74.1	20.7	5.2
	95.9	>99.9	>99.9	74.2	20.6	5.2
	94.7	>99.9	>99.9	73.9	20.9	5.2
NG9	97.6	>99.9	>99.9	69.5	20.3	10.2
	94.2	>99.9	>99.9	68.8	20.8	10.4
	94.9	>99.9	>99.9	68.9	20.7	10.3
NG10	94.2	>99.9	>99.9	63.7	20.8	15.6
	93.1	>99.9	>99.9	63.3	21.0	15.7
	89.4	>99.9	>99.9	62.4	21.5	16.1

Supplementary Figures

Figure S1. ^{1}H-NMR spectra for NG1-10 before (black, t = 0 h) and after polymerization (red, t = 24 h). Peaks integrated to obtain monomer conversion data are indicated with arrows and associated with the chemical structure of the relative monomer, crosslinker or internal standard.

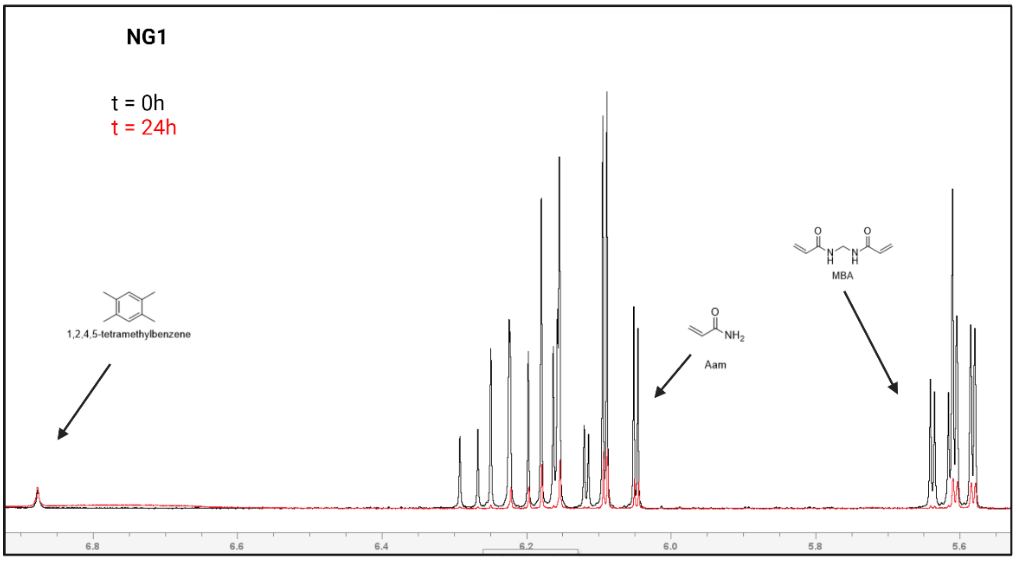

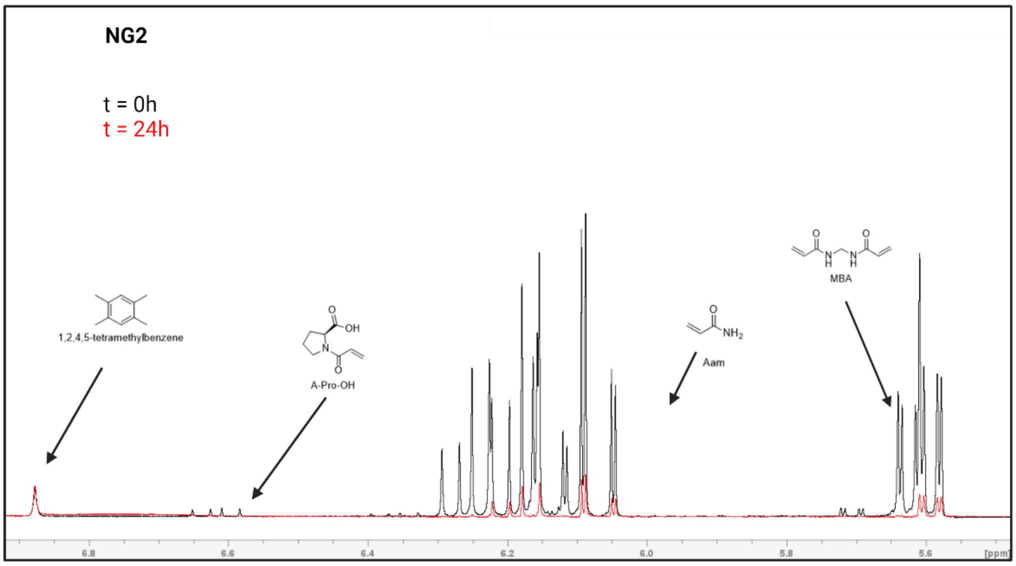

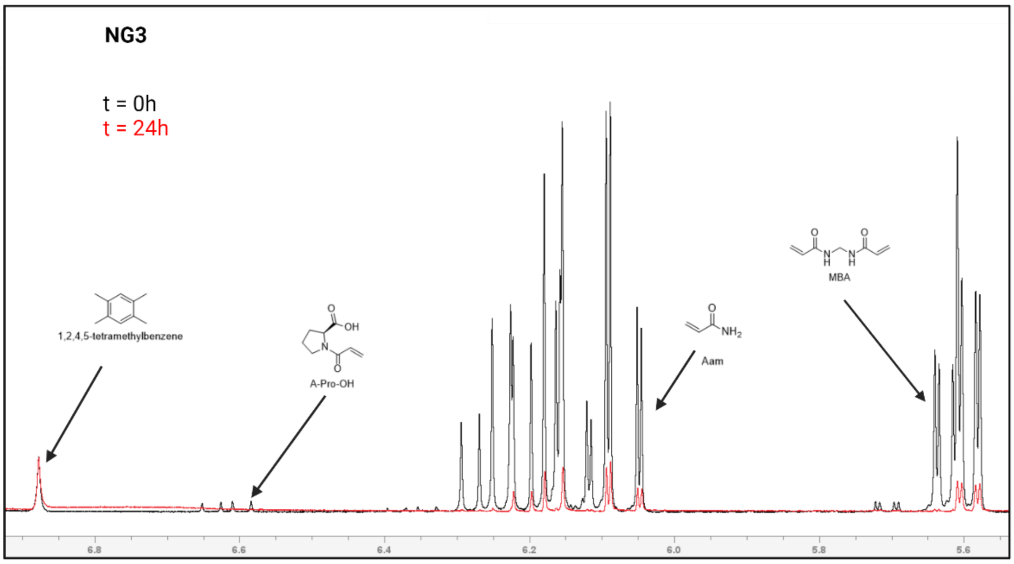
NG3
t = 0h
t = 24h
1,2,4,5-tetramethylbenzene
A-Pro-OH
Aam
MBA
6.8
6.6
6.4
6.2
6.0
5.8
5.6

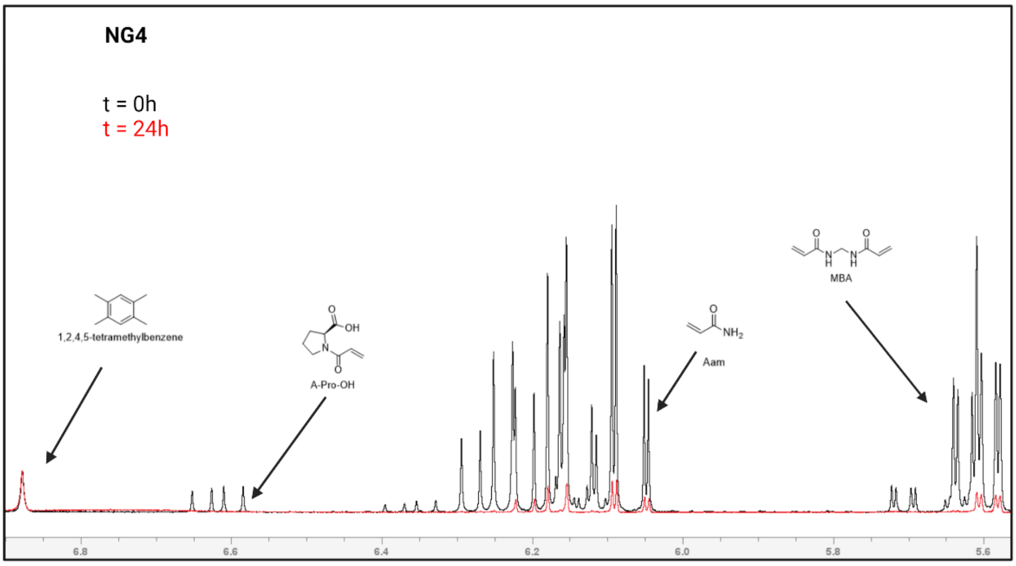
NG4
t = 0h
t = 24h
1,2,4,5-tetramethylbenzene
A-Pro-OH
Aam
MBA
6.8
6.6
6.4
6.2
6.0
5.8
5.6

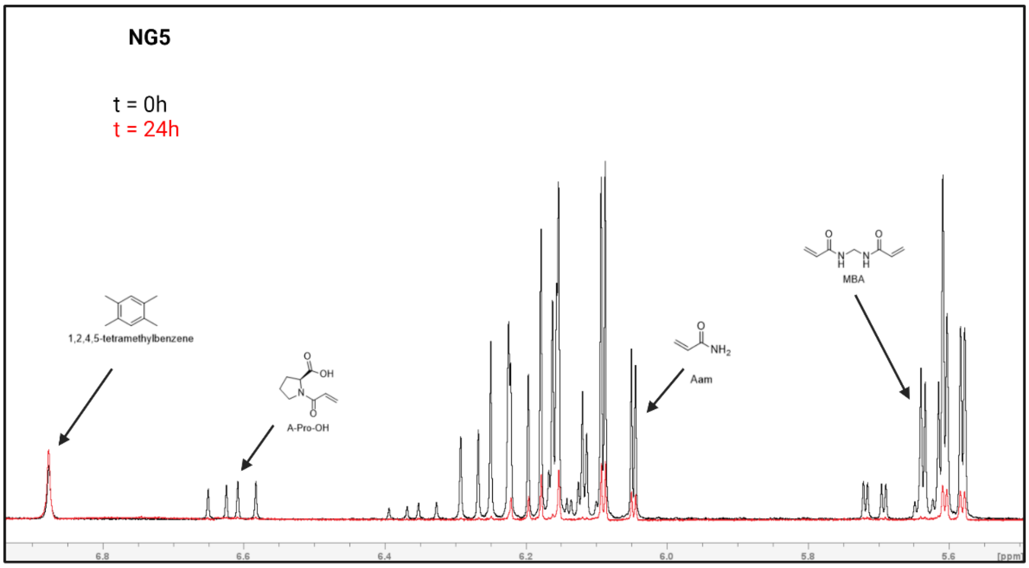
NG5
t = 0h
t = 24h
1,2,4,5-tetramethylbenzene
A-Pro-OH
Aam
MBA
6.8
6.6
6.4
6.2
6.0
5.8
5.6
[ppm]

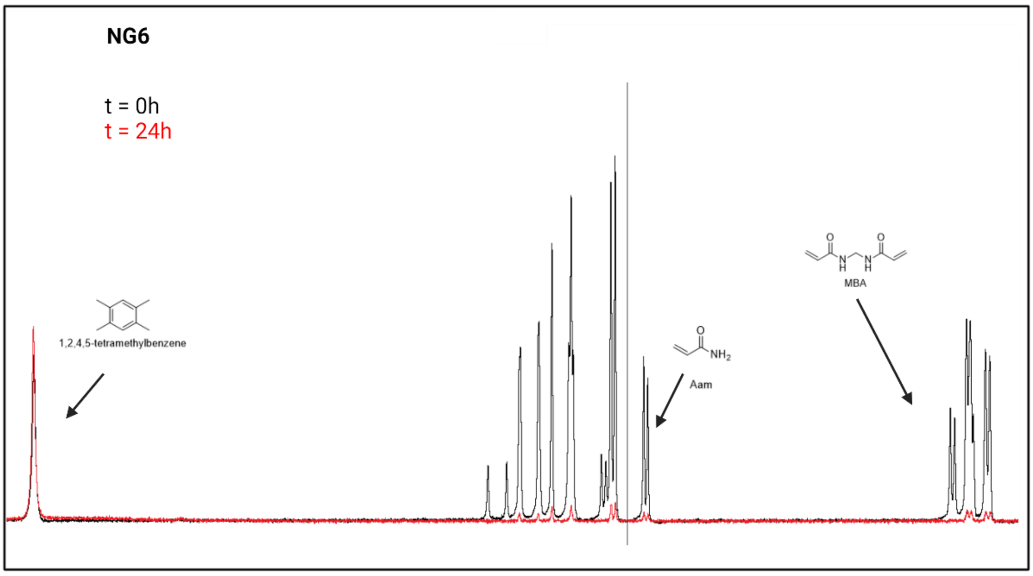

NG6
t = 0h
t = 24h
1,2,4,5-tetramethylbenzene
Aam
MBA

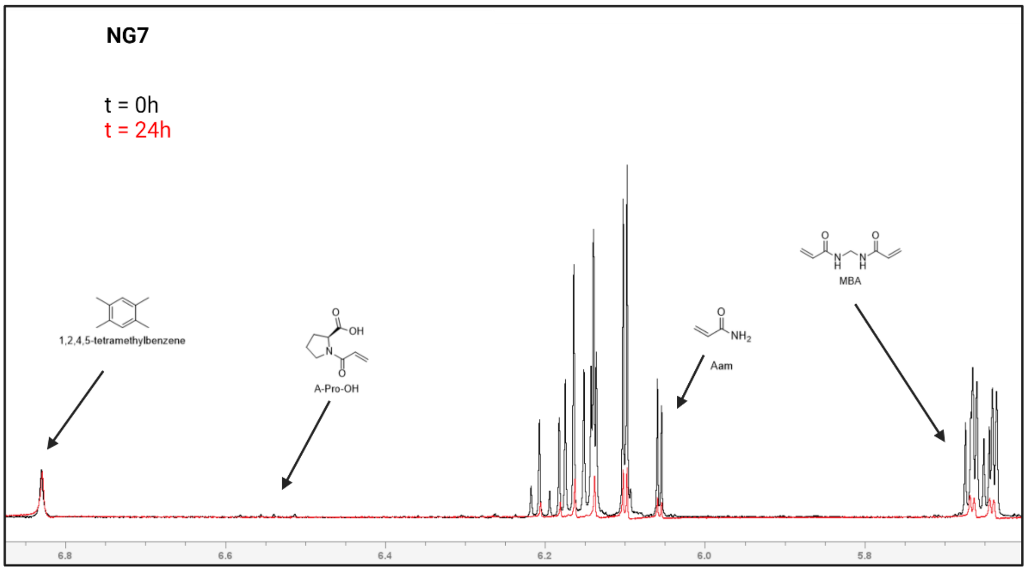

NG7
t = 0h
t = 24h
1,2,4,5-tetramethylbenzene
A-Pro-OH
Aam
MBA
6.8
6.6
6.4
6.2
6.0
5.8

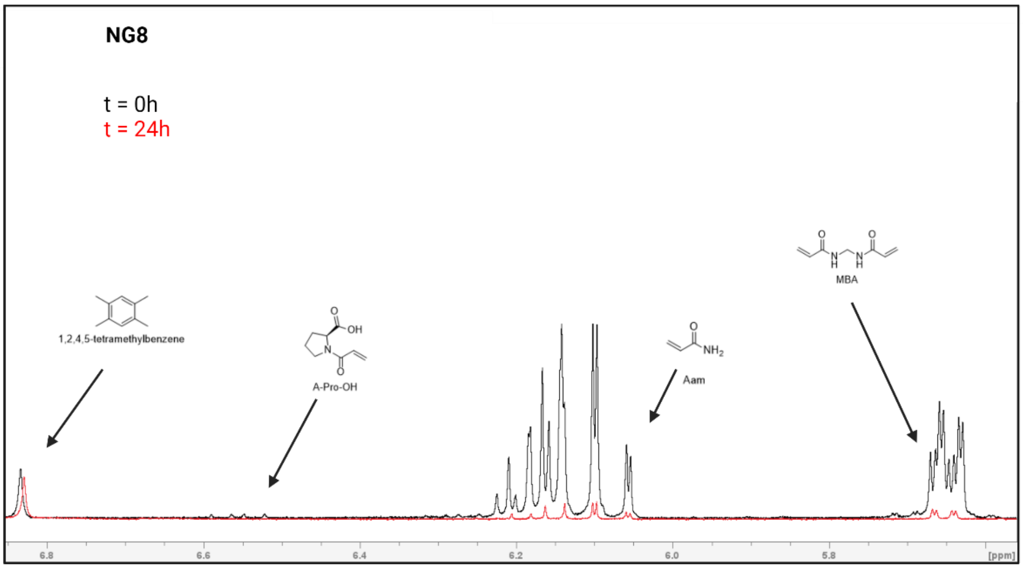
NG8
t = 0h
t = 24h
1,2,4,5-tetramethylbenzene
A-Pro-OH
Aam
MBA
6.8
6.6
6.4
6.2
6.0
5.8
[ppm]

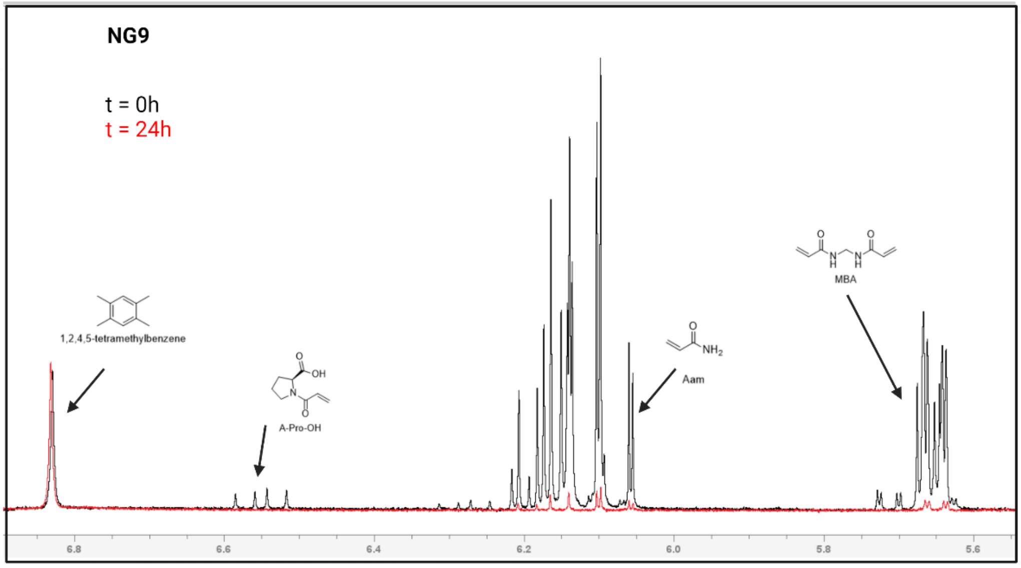
NG9
t = 0h
t = 24h
1,2,4,5-tetramethylbenzene
A-Pro-OH
Aam
MBA
6.8
6.6
6.4
6.2
6.0
5.8
5.6

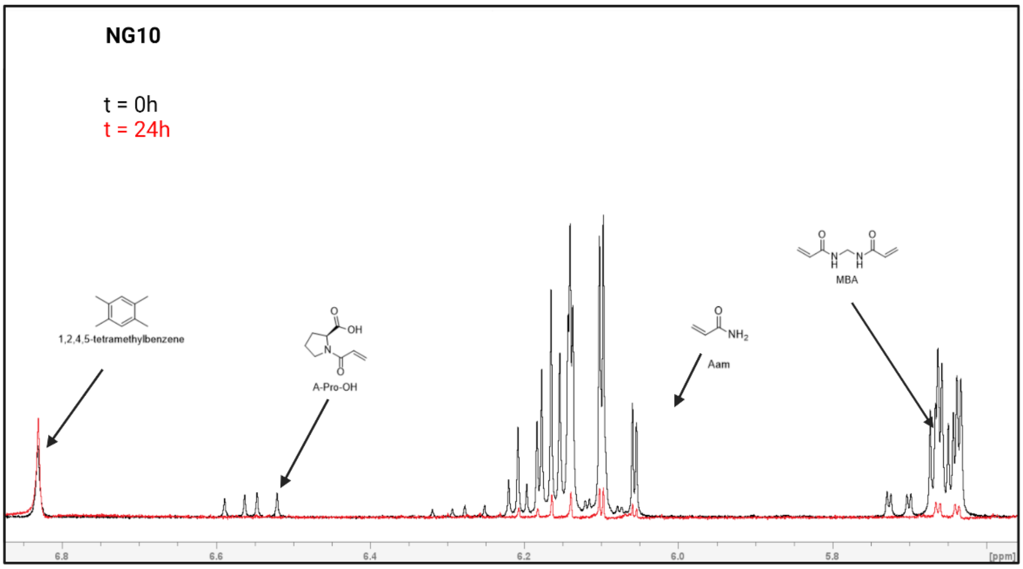
NG10
t = 0h
t = 24h
1,2,4,5-tetramethylbenzene
A-Pro-OH
Aam
MBA
6.8
6.6
6.4
6.2
6.0
5.8
[ppm]

Figure S2. Dynamic Light Scattering data and correlograms for NG 1-5 synthesized in DMSO. Data are plotted in total % distributions by intensity (blue), volume (violet) and number (pink) for each formulation.

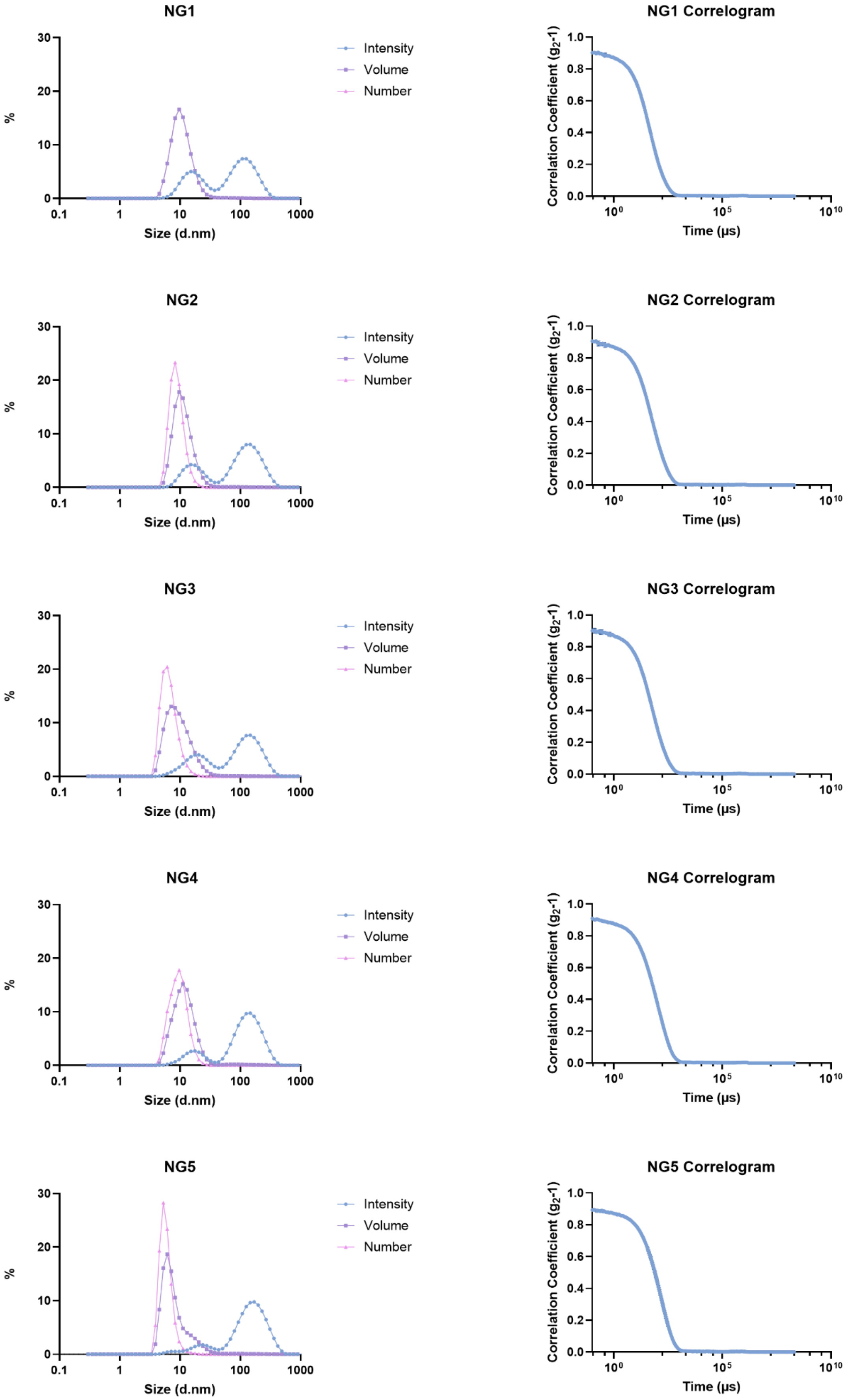

Figure S3. Dynamic Light Scattering data and correlograms for NG 6-10 synthesized in water. Data are plotted in total % distributions by intensity (blue), volume (violet) and number (pink) for each formulation.

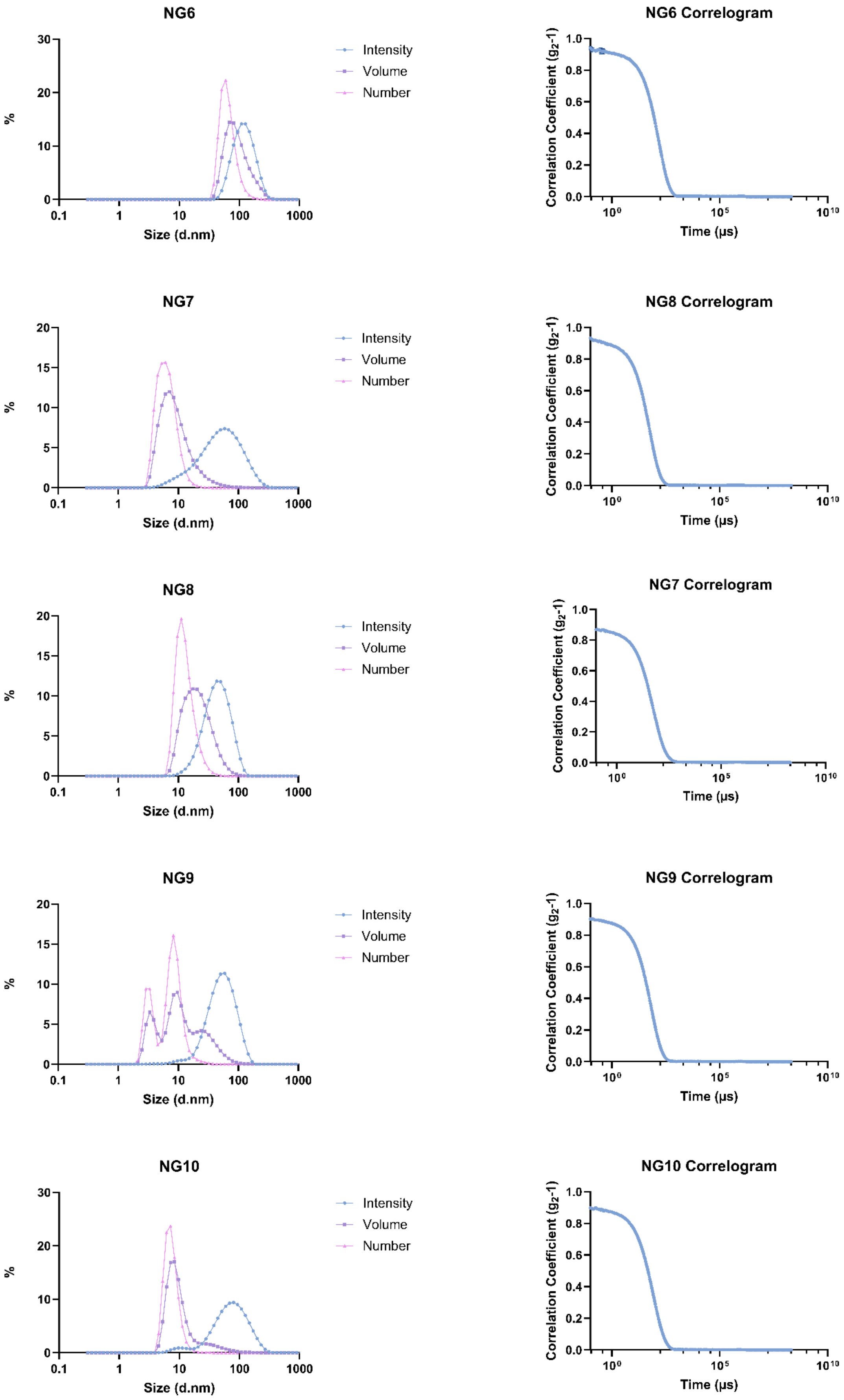

Figure S4. Cell viability expressed as percentage of medium control for different nanogels containing 0 to 10 molar% A-Pro-OH (vehicle control in orange, DMSO in grey, water in light blue). Concentration of NGs used is 0.5 mg/mL (a) and 0.1 mg/mL (b). Data are presented as mean ± SD (n=3).

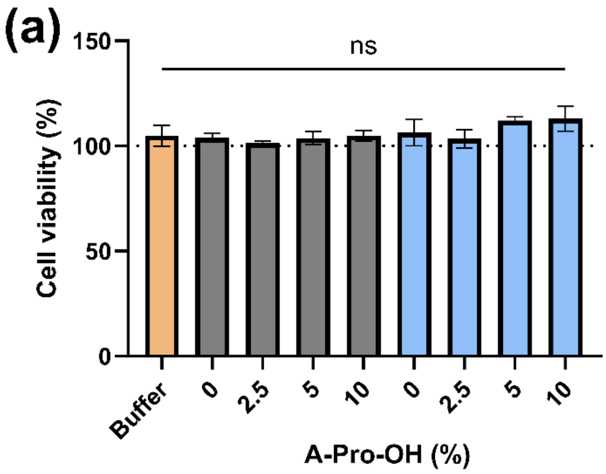

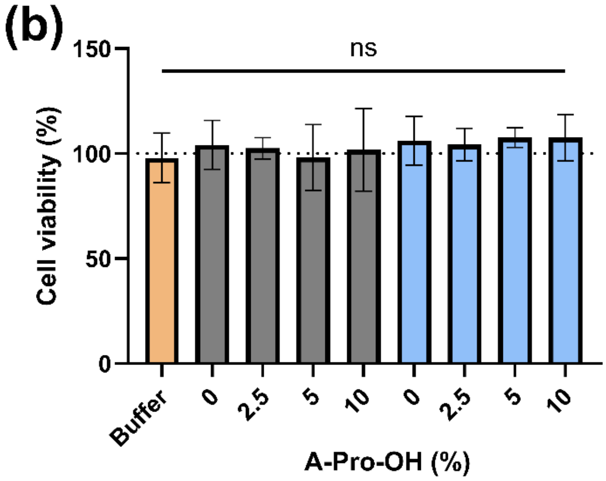

Figure S5. Negative staining TEM images for different nanogels containing 0 and10 molar% A-Pro-OH, synthesised in both DMSO (top) and water (bottom). Average diameter comparison of the formulations in both solvents is presented on the right-hand side as mean ± SEM (n = 105, **** $p < 0.0001$).

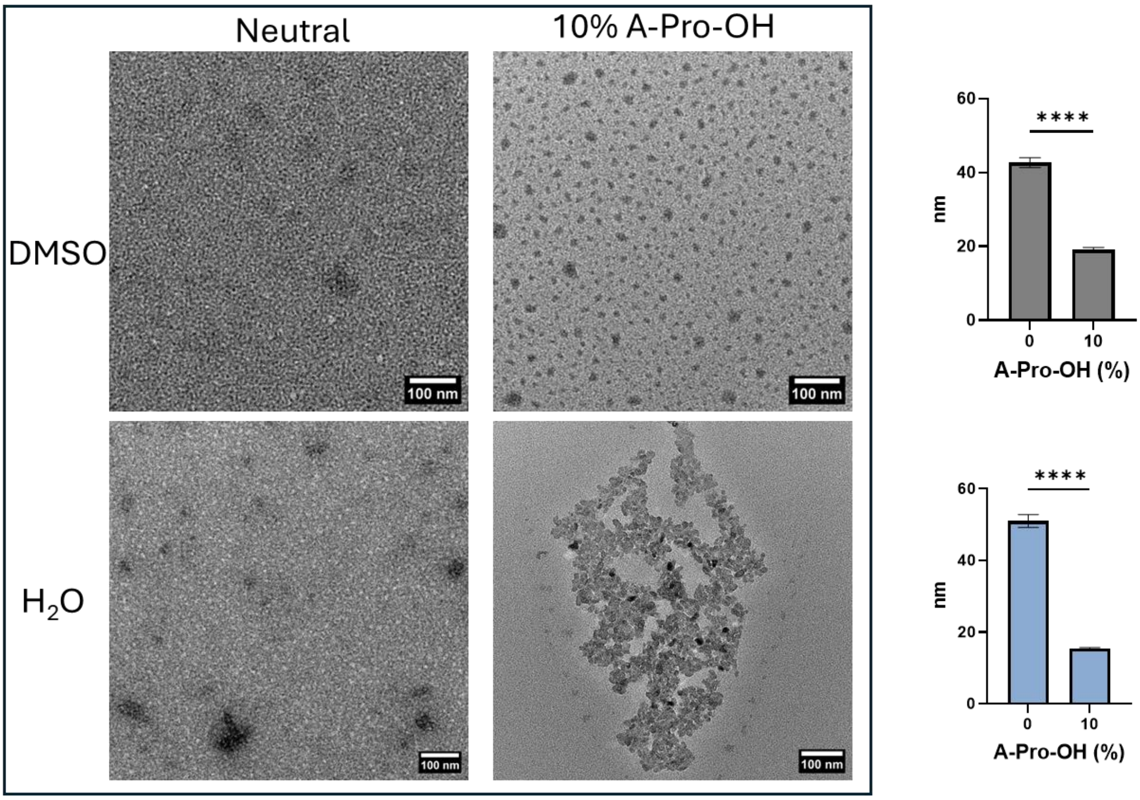

Figure S6. Cryogenic TEM images for nanogels containing 0 (a) and10 (b) molar% A-Pro-OH, synthesised in DMSO. Average diameter comparison of the formulations is presented as mean ± SEM (n = 105, * p < 0.01).

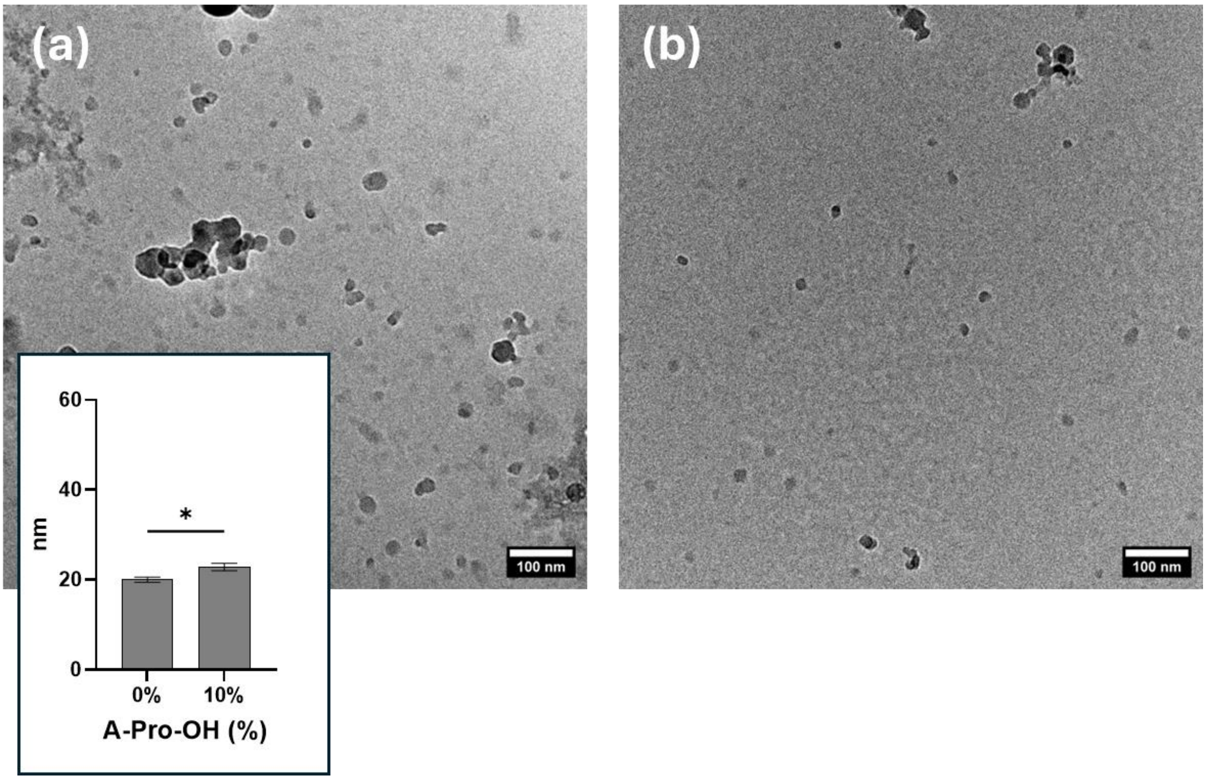

Supplement: Supplementary file 1 — ma4c02128_si_001.pdf [file ma4c02128_si_001.pdf]
